# Fibrohistiocytoma combined with an aneurysmal bone cyst at T7 in a 63-year-old woman

**DOI:** 10.3892/etm.2013.1272

**Published:** 2013-08-27

**Authors:** QI LI, YISHAN FU, YANG DONG, BINGFANG ZENG, CHANGQING ZHANG

**Affiliations:** Department of Orthopaedic Surgery, Shanghai Sixth People’s Hospital, Jiaotong University, Shanghai 200233, P.R. China

**Keywords:** fibrohistiocytoma, aneurysmal bone cyst, spine tumor, surgical procedure

## Abstract

We present a case of spinal tumor, with fibrohistiocytoma combined with aneurysmal bone cyst (ABC) at the pedicle and transverse process of T7 in a 63-year-old female. ABC is a rare skeletal tumor and spinal ABC is extremely rare. Fibrohistiocytoma is a type of primary benign bone tumor. ABC is also a rare bone tumor that most often occurs in the pelvis. The combined lesion of two primary bone benign tumors is relatively rare in clinic. In addition, fibrohistiocytoma and ABC are widely confused with other giant cell containing tumors of the bone. X-rays, magnetic resonance imaging (MRI) and positron emission tomography-computed tomography (PET-CT) scans were performed and assessed. Finally, the diagnosis was confirmed by pathological tests. The patient underwent surgery and had an extremely good recovery. The correct diagnosis of a spine tumor is important when determining the surgical procedure.

## Introduction

An aneurysmal bone cyst (ABC) is a rare skeletal tumor that accounts for ~1% of all bone tumors. A spinal location for an ABC is extremely rare. Fibrohistiocytoma is a type of primary benign bone tumor, which is composed of fusiform fibroblasts (1,2). It is a rare bone tumor, whose predilection site is the pelvis. They are more common in male patients. The combination of two primary bone benign tumors is quite rare in the clinic. From a diagnostic standpoint, fibrohistiocytoma and ABCs are widely confused with other giant-cell-containing tumors of the bone. Among such tumors, telangiectatic osteosarcoma is widely confused with ABCs, radiologically and pathologically, and there have been reports of telangiectatic osteosarcoma that were initially treated as ABCs with a fatal outcome (3). Therefore, the differential diagnosis between the two diseases should be conducted even more cautiously to prevent misdiagnosis.

## Case report

**Figure 1.**
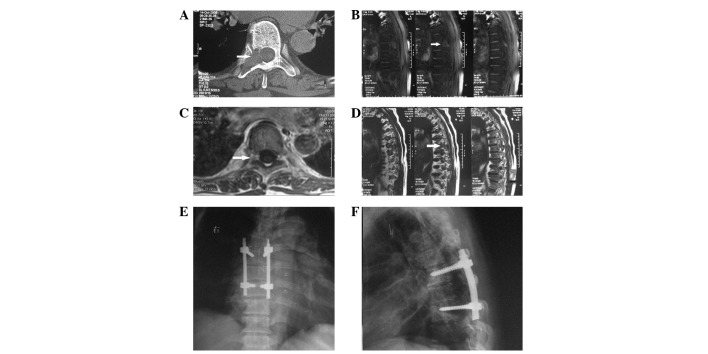
63-year-old female patient with an expansive, lytic and unicameral cyst lesion located in the right appendix of T7 (arrows). (A and B) CT scan and T2-weighted MRI imaging prior to surgery revealed an expansive, lytic and unicameral cyst lesion located at T7. (C and D) Sagittal fat-restrained and T1-weighted MRI revealed a highly hydrated lesion located at T7. (E and F) Anterior-posterior and lateral view X-ray post-surgery. Segmental instrumented posterior fusion from T6–T8 was performed after the tumor was removed. CT, computed tomography; MRI, magnetic resonance imaging.

**Figure 2.**
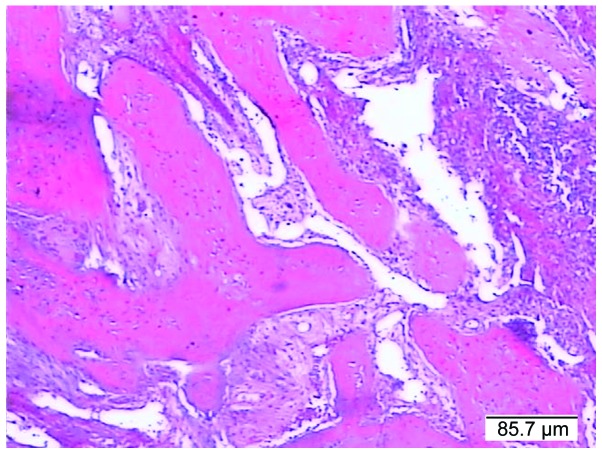
Histopathological examination (H&E; original magnification, ×40). Cyst-like changes and internal hemorrhage were observed within the cyst. The wall was composed of giant cell granulation tissue and responsive hyperosteogeny. H&E, hematoxylin and eosin.

**Figure 3.**
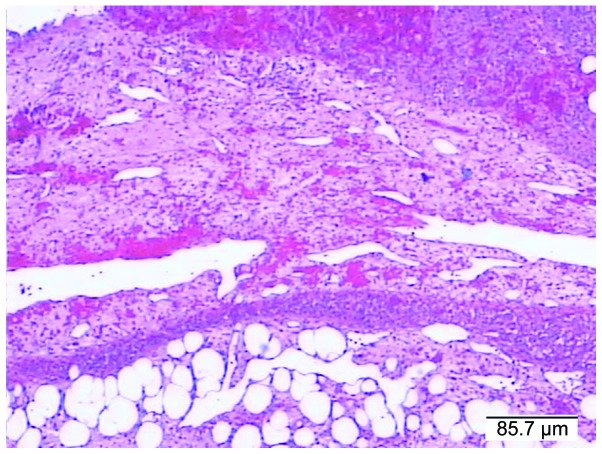
Histopathological examination (H&E, original magnification ×100). The endothecium of the cyst was giant cell granulation tissue and the exothecium was responsive bone. The responsive bone-like tissue was surrounded by benign osteoblasts. Additionally, cystose cells were surrounded by responsive hyperosteogeny, among which scattered inflammatory cell infiltration was observed. H&E, hematoxylin and eosin.

A 63-year-old female complained of back pain, and numbness and weakness in the lower limbs. Computerized tomography (CT) and magnetic resonance imaging (MRI) (Fig. 1A–D) revealed an expansive, lytic and unicameral cyst lesion localized at the pedicle and transverse process of T7. A nuclide bone scan revealed multiple nuclide aggregation in the costal bone and body of the vertebra. It was considered that the patient suffered from a metastatic tumor. However, after a thorough general examination, no sign of a primary tumor was identified. Then, the patient underwent a positron emission tomography (PET)-CT scan. Expansive osteoclasia was observed in the right appendix of T7, similar to a giant cell tumor of bone. Since the patient had rheumatic heart disease for ~10 years, heart ultrasound (heart shadow enlargement with back flow) was performed to evaluate heart function (grade II), to ensure the patient was able to endure surgery. Based on the clinical and diagnostic imaging findings, plans were made to treat the lesion with resection. Informed consent was obtained from the patient regularly. Prior to surgery, a puncture biopsy was performed, which resulted in the formation of scar tissue. Surgery was subsequently performed with the use of a general anesthetic, with the patient in the prone position. The tumor was resected and electrocautery was used following resection to prevent recurrence. Following excisional biopsy, segmental instrumented posterior fusion was performed from T6 to T8 (Fig. 1E and F). The surgery lasted for ~2.5 h. The blood loss was ~600 ml and 2 U red blood cells were transfused intraoperatively to maintain the normal blood volume. The pathological diagnosis was fibrohistiocytoma combined with an ABC (Figs. 2 and 3). Immunohistochemical analysis was negative for cytokeratin (CK), epithelial membrane antigen (EMA), CD34, CD31, desmin (Des), smooth muscle actin (SMA), HMB45, phosphoglucomutase 1 (PGM1) and S-100. The patient was followed-up for >2 years. The bone grafts had been incorporated and the patient was fully rehabilitated and free of any symptoms. Finally, the patient succumbed 5 years after surgery from epilepsy.

## Discussion

ABCs are benign, highly vascular osseous lesions characterized by cystic, blood-filled spaces surrounded by thin perimeters of expanded bone. The precise pathogenesis of ABC is unclear, although theories such as post-traumatic boney alteration, reactive vascular malformation and genetic predisposition have been described. The most widely accepted pathogenic mechanism for the development of ABC involves a hypothetical local circulatory disturbance that leads to markedly increased venous pressure and the development of dilated and enlarged vascular elements within the affected bone (3).

Fibrohistiocytoma is a type of primary benign bone tumor, which is composed of fusiform fibroblasts accompanied by multinucleated osteoclast-like giant cells, cystose cells and chronic inflammatory cell infiltration. There is also likely to be interstitial substance hemorrhage and hemosiderin deposition. It is a rare bone tumor and the predilection site is the pelvis. It is more common in male patients (4).

A combined lesion of two primary benign bone tumors is quite rare in the clinic (3). From a diagnostic standpoint, both fibrohistiocytoma and ABC are widely confused with other giant-cell-containing tumors of bone (3). Giant cell tumor (GCT), for instance, is composed of mononuclear cells and osteoclast-like multinucleated giant cells, with the potential to be locally aggressive. Histologically, a GCT consists of homogeneous stroma with giant and mononuclear cells dispersed evenly throughout the tumor. Giant cell reparative granuloma (GCRG) is an uncommon, benign, intraosseous reactive lesion for which the roentgenographic and histological features may overlap with ABCs. Brown tumors or von Recklinghausen’s disease typically involve the diaphysis of long bones. The incidence of brown tumors is reported to be 1.5–1.7% of patients with chronic renal deficiency. The radiographic and histological features of brown tumors, ABCs and GCRG are often indistinguishable (3). However, brown tumors were recently reported to have a much more oblated architectural growth pattern in comparison with ABC and GCRG. Hyperparathyroidism may be ruled out on the basis of serum calcium, phosphorus and parathyroid hormone levels.

Telangiectatic osteosarcoma is primarily composed of multiple dilated multi-cameral capsular cavities that contain blood, with viable high-grade sarcomatous cells in the peripheral rim and septae around these spaces. Hence, the lesion is widely confused with ABCs, radiologically and pathologically, and there have been reports of telangiectatic osteosarcomas that were initially treated as ABCs with a fatal outcome. Radiographic distinction between these lesions is challenging (3). However, the nodular viable high-grade sarcomatous cells that produce osteoid around these cystic spaces are demarcated following the administration of contrast material. The lesion appears as a solid, thick, nodular-enhancing rim of tissue that contains subtle mineralization when viewed with radiographs or CT images.

Grossly, an ABC is multiloculated, consisting of multiple blood-filled cystic spaces separated by thin, tan-white areas that may represent a solid portion of the ABC or a primary lesion, if present. Histologically, an ABC is composed of blood-filled cystic spaces separated by fibrous septae.

Methods for the treatment of an ABC include resection, curettage, embolization and intralesional injection of a variety of agents. In the current study, we present a female patient with thoracic spinal pain, with progressive paresthesias and muscle weakness of the lower extremities. This case highlights the importance of considering an ABC in the differential diagnosis of primary spinal tumors. The differential diagnosis for this lesion may be challenging, particularly with regard to the possibility of the presence of osteoid osteoma, osteoblastoma, ABC, osteochondroma, neurofibroma, eosinophilic granuloma, hemangioma and other giant-cell-containing tumors of bone, including GCT, GCRG, brown tumor of hyperparathyroidism and telangiectatic osteosarcoma. The evaluation of spinal tumors includes a thorough history assessment, physical examination, imaging and, occasionally, laboratory evaluation and biopsy when indicated (1).

Depending on the analysis of radiographic morphology, location and the age of the patient with ABC and GCT (5), the following criteria suggest an ABC with a high positive predictive value: location in the diaphysis and shaft, patient aged <17 years and growth rate grade Lodwick-IA. GCT was selected via the following criteria: epimetaphyseal location and growth rate grade Lodwick-II. However, in certain cases, differential diagnosis between the two entities is radiologically impossible.

In patients who develop a bone tumor of the posterior elements of the spine, careful clinical and radiological evaluation is necessary to narrow the differential diagnosis. Treatment options for ABCs are simple curettage with or without bone grafting, complete excision, embolization, radiation therapy or a combination of these modalities. Embolization appears to be the first option for spinal ABC treatment due to having the best cost-to-benefit ratio. An intact ABC is indicated when no pathological fracture or neurological involvements are observed (2). New therapies have recently been reported. Denosumab is a human monoclonal antibody that inhibits osteoclast function by blocking the cytokine receptor activator of the nuclear factor-κB ligand. Satisfactory results with denosumab in treating GCTs and immunohistochemical similarities suggest that it may also have positive effects on ABCs (6). Nevertheless, radical surgical excision should be the goal of surgery to reduce the recurrence rate. The recurrence rate is significantly lower in cases of total excision (7). In the majority of cases, a complete excision should be performed if possible. The risk of postlaminectomy kyphosis is high. As such, a fusion should be considered whenever a laminectomy is performed (8,9). Prompt detection and treatment with curettage, decompression and fusion produces a satisfactory result and prevents spinal cord injury (10). ABCs are hypervascularized lesions. Pre-operative selective arterial embolization of the lesion may have some value (11). Selective arterial embolization should be used as a preoperative adjunct to surgery for ABC of the spine to reduce intraoperative blood loss and the need for blood transfusions. Spinal fusion with titanium instruments (12) is strongly recommended if more than two facets (more than one on each side, or more than two full facets) are violated during the excision of the ABC.
